# Assessing factors militating against the acceptance and successful implementation of a cloud based health center from the healthcare professionals’ perspective: a survey of hospitals in Benue state, northcentral Nigeria

**DOI:** 10.1186/s12911-019-0751-x

**Published:** 2019-02-19

**Authors:** Patience E. Idoga, Mehmet Toycan, Halil Nadiri, Erbuğ Çelebi

**Affiliations:** 1 Management Information System Department, School of Applied Sciences, Cyprus International University, via Mersin 10, Nicosia, North-Cyprus Turkey; 2 Business Administration Department, Cyprus International University, via Mersin 10, Nicosia, North-Cyprus Turkey; 3 Department of Computer Engineering, Cyprus International University, via Mersin 10, Nicosia, North-Cyprus Turkey

**Keywords:** Technology acceptance, Healthcare professionals, UTAUT2, Cloud computing, Nigeria

## Abstract

**Background:**

Cloud based health platforms (CBHP) have tremendous capacity to meet patient’s health needs. The benefits inherent in CBHP position it to be relevant for efficient healthcare delivery. Nonetheless, studies have shown that the adoption of new technologies is sometimes a challenge especially in developing nations. This study, therefore, aim to examine, identify and evaluate the factors affecting healthcare professionals’ intention to accept the cloud-based health center (CBHC) in developing countries. The research study focuses on hospitals in North-central of Nigeria.

**Methods:**

Using questionnaire adopted from related studies, a cross-sectional study was carried out of 300 healthcare professionals selected from medical health institutions in Benue State Nigeria. The study adopted the Unified Theory of Acceptance and use of Technology Extended (UTAUT2). Data analysis was carried out using SPSS (V20.0) and LISREL (V9.30) generally employed in Structural Equation Modeling to examine components and path model. The Socio technical design method was used to develop the CBHC.

**Results:**

Findings portrays performance expectancy, cloud based health knowledge, IT infrastructure and social influence to have significant effects on the intentions of healthcare professionals to accept and use the CBHC. These findings, agrees with prior related studies.

**Conclusions:**

Our findings impacts the body of knowledge in that it identifies important areas the studies can be useful, especially, to managers and healthcare policy makers in the planning/implementation of health cloud. Research findings from the theoretical acceptance model identifies the factors and barriers towards sustainable cloud based health center solutions to meet the healthcare needs of people in remote communities.

## Background

It is an important strategy both for the government and healthcare industry to provide efficient healthcare delivery while planning to utilize the latest technologies. Global human population has shown an enormous increase in the recent years and diseases are becoming more complex. As a result, new advancements in technology have exploited in order to provide more effective diagnoses and treatment techniques while reducing the operations costs [1]. One of the ways this can be achieved and the health care system made robust is through the adoption of cloud computing into the health care system. Cloud computing has evolved over the years from basic storage platforms to encompass more comprehensive solutions for on demand delivery over the Internet, such as handling big data [2].

In the healthcare domain, healthcare service providers are integrating cloud based health platforms (CBHP) to reduce cost of operation, loss of data, prompt/remote access to data, etc. thereby, bringing efficiency both to the system and patients healthcare delivery. Using CBHP ensures that patient’s data are readily available, lost data can be recovered, transfer of patients clinical records is made possible and all clinical data are integrated into a central location for easy access for authorized persons [3]. It capabilities also includes, prompt/easy access to data and information [4] and IT resource sharing [5, 6]. Cloud computing proffers the resource sharing option for healthcare service providers with the other stakeholders such as other hospitals, government agencies etc. While sharing could improve the quality of service for the patients, it should also have strict regulations for privacy and data security. The benefits inherent in CBHP position it to be adequate for efficient healthcare service delivery most especially, for developing countries who, according to studies, are yet to meet up with twenty first century health care delivery needs. Studies have shown that CBHP present a good ground for coordination between all healthcare workers [7]*,* connections [8] more importantly, aid the transfer of clinical health records [9]*.* It was revealed from the previous studies that IT literacy and experience of healthcare service providers have a significant influence on their perception, attitude and intention for the use of a new technology such as e-health technology applications [10, 11]. In accordance, Cloud based health knowledge (CBHK) of CBHP by users, is considered necessary for the sustainability of cloud-based healthcare solutions. It would be easier for the healthcare service providers to use cloud based health care systems if they have a prior knowledge of the systems’ working environment [11].

The World Health Organization (WHO) noted that, a robust healthcare system is one which provide adequate healthcare services to meet the health needs of people in a certain geographical location whenever needed [12]. With this understanding, the term cloud based health system refers to all computing mechanisms such as infrastructure, software and hardware used by healthcare service providers to deliver healthcare services like Software as a Service (SaaS), Infrastructure as a Service (IaaS) and Platform as a Service (PaaS) [13]. The factors mentioned above has been considered in the design of cloud based computing systems for the care of elderly and patients with anxiety disorders [14], patients with chronic diseases [15] and monitoring of patients with artificial hearts [16].

Health institutions in Nigeria remain under developed not mainly because of inadequate government funds, but also due to inadequate utilization of the available funds in the areas of appropriate focus on urban/rural health information and communication technologies (ICTs) [17]. In order to curtail this, developing countries such as Nigeria need to harness the potentials of cloud computing in their health care system by using cloud solutions such as: (1) SaaS: the mechanism in which software applications are provided over the internet by service providers, (2) PaaS: the mechanism in which computing solutions stacks and platforms are accessed by IT managers, developers or users over the internet without having to download any software and (3) IaaS: the sharing of computing resources through a virtualized environment) to bring about reducing cost, service speed and the elimination of inadequate health care workers [13, 18].

Currently, the national health care system in Nigeria is divided into three tiers which are: the primary health care (PHC), the secondary health care (SHC) and the tertiary health care (THC). All operating on decentralized systems and burdened with the tasks of providing effective health care services/programs to the citizens. The federal government specifies the health care polices and ensures that these policies are safeguarded by the PHC, SHC and THC [19]. While the THC is well equipped with adequate internet facility along with good source of energy supply and other infrastructures, the PHC and the SHC have limited or no access to the internet, and energy supply is even not providing the necessary quality of service. However, the increased use of internet enabled mobile devices and other means of energy supply (e.g. power generator) have motivated people not to depend on the public electrical and internet service authorities, since they can subscribe privately from private service providers (e.g. Glo-mobile network, MTN, Etisalat, Airtel etc.). Recently in November 2017, the Nigerian Communication Commission (NCC), revealed that 98.3 million Nigerians are internet users. Similarly, the International Telecommunication Union (ITU) 2017 report of Nigeria, identified a 75.9% mobile-cellular subscription users per 100 populations. Signifying a surge in the use of mobile–cellular subscription as against 33% in 2013 [20].

The introduction of new technologies is a very important development; more important is its application in the field of healthcare. These technologies enhance the efficiency both for healthcare professionals and patients with quicker and better access to healthcare services; but also facilitates proper and accurate decision making for stakeholders [21]. Despite the potentials inherent in the use of the CBHS to bring about effective and efficient healthcare service delivery to remote/rural communities in developing countries, research studies [22] have shown that its acceptance and usage is still a challenge, especially among healthcare professionals in some developing countries. Resulting in a gap between implementing a technology and adopting that technology. There is, therefore, need for healthcare professionals to accept the use of these technologies as their acceptance is pivotal and highly relevant to the success and sustainability of the systems [23]*.*

It has been revealed from the studies [24] that before some of these technologies are accepted and utilized, they are required to meet up with some expectations of users’ requirements. Hence, different theoretical models have proposed to realize the factors and perceptions of technology acceptance. Popular among them are: (1) Theory of Reason Action (TRA): this theory explains the connection between behavior and attitudes in the context of human action. Its objective is to predict the intention why a behavior is performed by leveraging on each person’s attitude and beliefs [25]. (2) Technology Acceptance Model (TAM 1 and TAM 2): TAM is one of the well-known information systems theory. It models the factors that influence the users’ decision when introduced to a new technology and their subsequent usage. Over the years, new variables such as integrating barriers and network effects as external factors were added to the TAM model so as to derive a better understanding for users’ intention to utilize a specific technology, resulting in TAM 2 [26, 27]. (3) Unified Theory of Acceptance and use of Technology (UTAUT and UTAUT2): is a model employed to assess the level of new technology acceptance by users. Due to some limitations relating to generalization in the original UTAUT, UTAUT2 was developed and tailored to suit the consumer context [28]. While TRA is a model which is good at the prediction of human behavior [29], TAM is designed to provide a theoretical framework for the factors that influences user’s actions to the usage of information systems [30] and UTAUT is modelled towards user’s motivation to use an information system as well as their future behavior [31]*.*

Developing countries such as Nigeria with a very large population are faced with a lot of challenges in their health care system: prevalent among these challenges are inadequate availability of healthcare professionals and medical health facilities among others as noted by [32]. As a result of this, we have people traveling long distances from remote rural villages to urban cities for their medical needs. This further result in excessive pressure on the part of healthcare professionals. Despite these challenges, studies have indicated that healthcare professionals in Nigeria are yet to adopt cloud-based health platforms, which could eliminate the issues enumerated [23]*.*

To this end, the primary research objective of this study is to assess the factors which have a significant effect on the adoption of cloud based health systems by healthcare professionals in Nigeria. In addition, a Cloud Based Health Center is proposed for remote care and prompt access to health care needs for the future use. Findings from the theoretical acceptance framework could enhance sustainable and successful implementation of a Cloud Based Health Center. This could provide remote service delivery to meet the healthcare needs of people in remote/rural communities in Nigeria. Hence, the main questions of the research are:

- What is the level of cloud based health knowledge (CBHK) of healthcare professionals in Nigeria?
- What are the factors that can militate against the acceptance and use of Cloud Based Health Systems (CBHS) in Nigeria?
- How can Nigeria adopt and use the CBHS?

Our research contribution could be seen in terms of the acceptance determination of healthcare professionals in using the cloud based health systems in Nigeria by using the guideline of [10] in verifying the use of the Unified Theory of Acceptance and use of Technology Extended (UTAUT2) in healthcare. Also, proposing the CBHC which can aid remote care to people in rural and remote communities. In addition, it is a crucial research objective to realize the effect of cloud based health knowledge (CBHK) as a major influence on the adoption and usage of cloud based health systems (CBHS) by the healthcare professionals. Our study investigated issues that mitigate either the acceptance or rejection of CBHS among healthcare professionals in Nigeria. Different factors are considered in the proposed model which influences behavioral intention to use CBHS. Our proposed system could be replicated and made to suit any organization which intend to adopt any cloud based systems.

## Cloud based health technologies and acceptance models

Cloud based health platforms (CBHP) have tremendous capacity to meet patient’s health needs if its potential are fully utilized. The cloud makes possible a lot of things since it is open to all making data access, storage, retrieval and computations easy and readily available since it has no physical localization [33]*.* The term cloud based used here refers to some set of centralized components and tools accessible from remote locations via the use of web browsers [34]. CBHP can handle big data as well as pictures and text, thereby presenting a comprehensive and up to date health database. As a result of this, healthcare professionals have prompt and remote access to clinical data/information which in turn enables them to take adequate medical decisions. The functions performed by the CBHP include, but not limited to the enhancement of health care services, disease identification, monitoring and treatment, self-management of diseases and so on [35]*.*

Health institutions were using the manual information processing and filing cabinet storages before the use of information technologies in healthcare delivery which leads to loss of patients’ data and even unnecessary waste of time in looking for a patient record [36]. It should also be noted that since the hospitals are not on the same platforms or using information sharing, referral is found to be very difficult once a patient is asked to go to another health institution. Hence, the use of cloud computing in healthcare could resolve all these issues such that access to patients’ records is not hindered by geographical location [37]*.*

There are several studies in the literature in which the acceptance and intention to use a cloud based healthcare technology solutions have been investigated [38–40]. On the other hand, medical doctors’ intention to use a clinical decision support system is studied in [41] while performance expectancy (PE), social influence (SI) and self-efficacy (SE) were realized as significant factors. PE can be defined as “the degree to which the user expects that using the system will help him or her to attain gains in job performance” [31]. On the other hand, SI can be defined as “the degree to which an individual perceives that important others believe he or she should use the new system” while SE can be defined as “the degree to which an individual skills are required to use the new system” [31]. Integrated TAM and UTAUT based research study in conducted in [42] in order to determine the level of e-health applications among German healthcare professionals. It was revealed from the study that both perceived usefulness (PU) and perceived ease of use (PEOU) have a positive influence on their intention to use the technology. PU is defined as the conviction and opinion of an individual that the use of a certain technology will increase their performance with better productivity. On the other hand, PEOU is defined as an individual belief that the use of a certain technology would free of effort [42]. Studies have shown that healthcare professionals’ acceptance of cloud based technologies differs when compared to healthcare consumers in the sense that their acceptance is dependent on the perceived usefulness of the technology rather than its ease of use [43]*.*

Some studies have investigated the attitude of doctors, nurses, lab technicians and other stakeholders as regard the acceptance of cloud based technologies (CBT) using either TAM or UTAUT. For instance, TAM is used in [44] to determine the factors which aid the acceptance of healthcare cloud computing technologies by healthcare professionals in Klang Valleys, Malaysia. On the other hand, Slade et al. [45] employed the UTAUT2 to investigate the motivating factors of user’s intention to use mobile technologies in the healthcare domain. In another study [46], factors were studied that predict the adoption and usage of Electronic Medical Records (EMRs) among family physicians using the UTAUT. It was found that SI and PEOU does not have a strong correlation as compared with PU, facilitating condition (FC) and general satisfaction (GS). The summary of cloud based technology acceptance can be found in Table 1.

**Table 1:** Summary of cloud computing acceptance

Author/year	Title	Subject	Model	Findings
Hsieh P.J (2016) [40]	An empirical investigation of patients’ acceptance and resistance toward the health cloud: The dual factor perspective	Patients	UTAUT	Findings show that patients acceptance or otherwise of a health cloud shows a negative effect.
Hsieh P.J. (2015) [83]	Healthcare professionals’ use of health clouds: Integrating technology acceptance and status quo bias perspectives	Health workers		Findings show a negative trend in the relationship that exist between health workers intention or otherwise to the usage of the health cloud.
Su Chen-Ying et al. (2015) [38]	Willingness of E-care Cloud System Using-Based on a Long-term Caring Institution in Southern Taiwan	Caregivers	TAM2	Findings show that caregivers are willing to use the E-care cloud system. In addition, information literacy and working association were seen as being able to enhance caregivers’ willingness to use the system.
Chen M.S. et al. (2016) [39]	The Intention to Use the Cloud Sphygmomanometer-Demonstrated by Taiwan Medical Center	Patients	TAM	Investigations identified specific important factors such as social influence, anchor and adjustment as major influence for using cloud sphygmomanometer and how this factors relates with TAM.
Sedem et al. (2015) [45]	Cloud Computing Framework for E-Health in Ghana: Adoption Issues and Strategies: Case Study Of Ghana Health Service	Healthcare professionals	Diffusion of Innovation Theory (DOI)	Findings indicate that most healthcare professionals in Ghana, are well informed with modern health technologies.

## Unified theory of acceptance and use of technology (UTAUT)

**Fig. 1:**
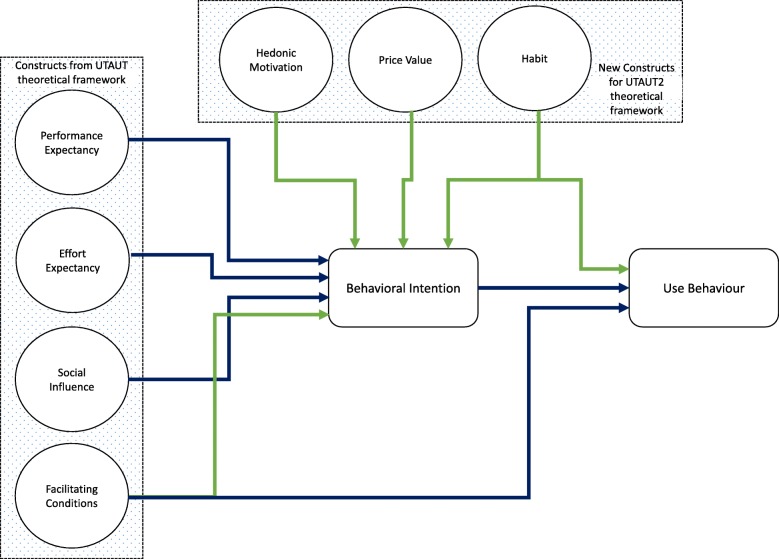
UTAUT2 theoretical framework (Source: authors)

The unified theory of acceptance and use of technology (UTAUT) is developed after reviewing several acceptance models and theories [22]. This model incorporates four basic construct which are: performance expectancy, effort expectancy, social influence and facilitating conditions. It is believed that while the first three constructs greatly influence behavioral intent, facilitating conditions and behavioral intention determine the actual use of a technology. This model also has some moderating variables such as gender, age, experience and voluntariness which show the relationships between the variables [46]*.* UTAUT2 theoretical framework is developed in time, as shown in Fig. 1, due to some limitations of UTAUT [10, 47]. New relationships in UTAUT2 model are shown in green color.

Three new constructs were added to the earlier four constructs, namely hedonic motivation, price value and habit in order to relate the model to consumer perspective. In accordance, UTAUT2 includes: performance expectancy, effort expectancy, social influence, facilitating conditions, hedonic motivation (seen as the enjoyment one derived from using a particular product), price efficacy (referred to as the cost a user incurred in order to use a particular service), and finally habit (seen as the extent of behavioral characteristics) [48]. It should be noted that voluntariness, which is a moderator in UTAUT, was dropped in UTAUT2 and facilitating condition is hypothesized to influence both behavioral intention and user behavior directly due to the factor’s critical role in an organization environment [10].

## Cloud based health center

**Fig. 2:**
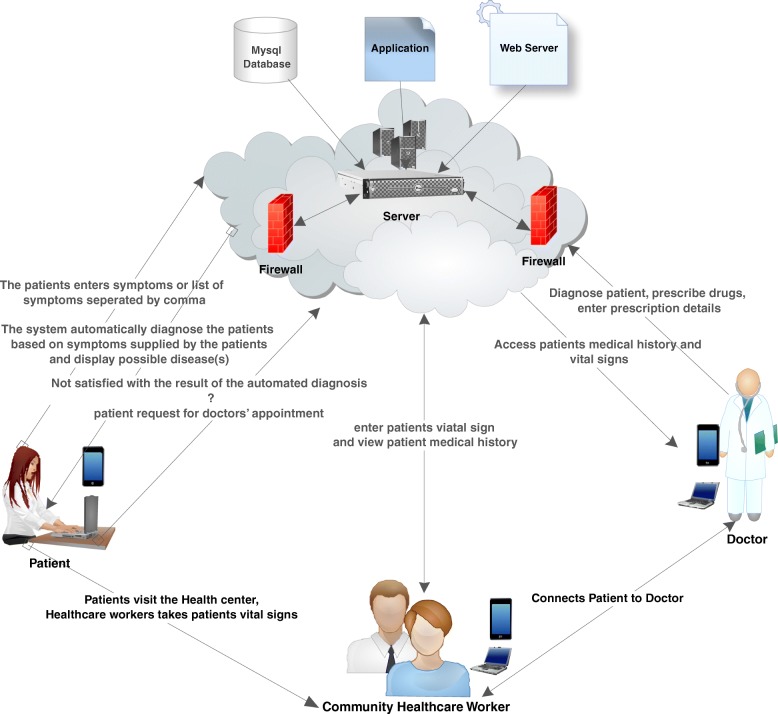
CBHC system architecture (source: Figure created using the demo version of ConceptDraw Diagram software which adheres to the terms and conditions of ConceptDraw)

Using a socio-technical design approach suggested by [49] in the design and development of health information technologies, Cloud Based Health Center (CBHC) is developed, as can be seen in Fig. 2. The CBHC is a web based application that enhance the provision of distant health care delivery for both healthcare professionals and healthcare consumers.

The system has majorly three main actors: the doctor, the healthcare worker (HCW) and the patient. The functions of the doctor include diagnosis, drug prescription and access to patient records and vital signs. The HCW however, is responsible for registering a new patient, check/enter a patient vital signs (body temperature, blood pressure, weight, etc.) and serve as an intermediary between the doctor and the patient in cases where a patient has an appointment with a doctor. After registering to the system, username and temporary password are generated which can also be changed in the future. Users could have the chance to view their medical results, enter the symptoms of a disease they are feeling in order to get a diagnosis. They can also book an appointment with a doctor in cases where a patient feels that the inputted symptoms is not adequate enough for accurate system diagnosis. It should be noted that the patients cloud log-in to their accounts by using their internet enabled devices, such as mobile phones, tablets, computers etc.

However, patients who do not have access to a personal IT device could also use the CBHC through the HCW who is stationed at the community health center. The system is protected by a firewall so as to protect and secure patients’ data and managed by a system administrator whose major responsibility is to register new doctors and HCW to the system.

A login system is utilized to access the application for all class of users. Based on the type of users, the system automatically, directs them to their respective interfaces. The system is developed to facilitate and enhance the working efficiency of health care professionals so as to relieve them from undue pressure, which might arise as a result of the limited number of healthcare professionals. The system is also designed to facilitate remote healthcare delivery through Telepresence: in that way, healthcare professionals don’t have to be physically present in order to attend to the healthcare needs of patients. In addition to the firewalls, an APACHE web server is used as a security measure to monitor the activities in the system providing a secured, efficient and extensible server with HTTP services in synchronization with recent HTTP standards.

The Nigerian National Health Act (2014) [50], offers a legal framework that regulates the administration and expansion of the National Health System and also, set criteria for the adequate delivery of health care services in the country. Under this Act, health technologies and institutions are categorized based on their functions to the national health care system. The type or level of health services are categorized with respect to the offered service type as well as the need to structure healthcare service delivery in line with national framework standards.

Cloud Based Health Center is developed in order to provide solutions to the issues, such as shortage of healthcare professionals and facilities as well as excessive pressure on healthcare professional. The proposed model could be used in the future to be of benefıt to both healthcare professionals and consumers.

## Research model and hypothesis

**Fig. 3:**
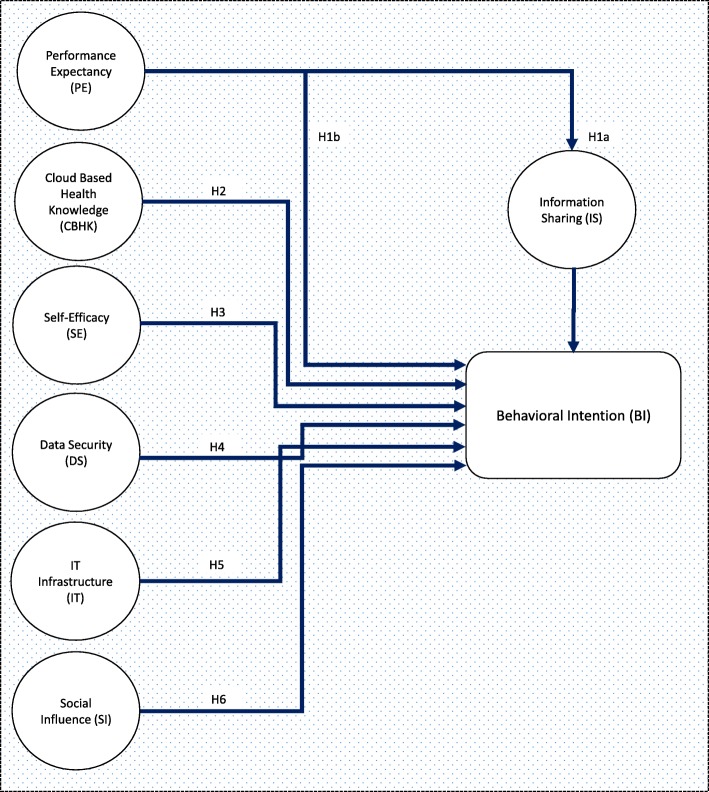
Hypothesized Research Model

Sequel to the discussion of UTAUT, an extended UTAUT model is used in this research to identify the factors which negate health care professionals’ acceptance of CBHC. Our research framework proposes new constructs which include Cloud Based Health knowledge (CBHK), data security (DS), IT infrastructure (IT) and information sharing (IS). It was shown in the previous studies [51, 52] that UTAUT2 theoretical framework has high abilities in explaining the behavior intentions of healthcare professionals at using healthcare information systems. In addition, it justifies other UTAUT2 findings in related healthcare context which confirms the ability of UTAUT2 to be able to predict the underlying factors to the acceptance of CBHC by healthcare professionals. The proposed hypothesized research model is depicted in Fig. 3 as regard the relevance of adoption and use of cloud based health center (CBHC) by healthcare professionals in developing countries.

Research constructs are explained as follows:

### Performance expectancy

Performance expectancy is termed the extent in which the use of a particular technology yield positive impact to consumers in the performance of named activities [52]*.* Similarly, recent research has held up the relevance of performance expectancy in user’s intention to accept a technology [53–55]. Therefore, performance expectancy has been hypothesized as a direct determinant of behavioral intention and also information sharing for data exchange support.

***H1a:*** *Performance expectancy is positively related to information sharing.*

***H1b:*** *Performance expectancy is positively related to healthcare professional behavioral intention to accept and use the health cloud.*

### Cloud based health knowledge

Knowledge of cloud based health platforms which is a motivating factor for the construct effort expectancy is seen to be a very essential requirement for using any cloud based systems as stated by [56]*.* The awareness and technological skills possessed by a healthcare service provider position them in an advantageous position to gain the maximum benefits attainable. It was revealed from the previous studies [44] that the awareness of cloud based applications in healthcare is lower in developing countries which could have an effect on the use of health cloud. Therefore,

***H2:*** *Cloud based health knowledge is positively related to health care professionals’ behavioral intention to use the health cloud*.

### Self-efficacy

The usage of the cloud based health center means that a user should have necessary skills which will enable the use of the proposed platform. Self-efficacy is considered as one of the facilitating conditions in which the necessary technical support is crucial for the use of a certain technology in system operation [29]. If the facilitating conditions are available, such as demos, CDs and online tutorials, on how to use cloud based health platforms, then health care professionals will have the intention to use the platform. Hence,

***H3:*** *Self-efficacy is positively related to healthcare professionals’ behavioral intention to use the health cloud*.

### Data security

Data security here refers to the measures applied to protect personal data and information from unauthorized access. The data security is a major issue in cloud based systems since unauthorized access can result in serious damage; hence the need for adequate regulations and preventive measures is imperative. It was revealed from previous studies that a robust association exists between data security and health care professionals’ intention to use cloud based health technologies [57]. Therefore,

***H4:*** *Data security is positively related to health care professionals’ behavioral intention to use the health cloud*.

### IT infrastructure

The availability of required ICTs (hardware and software) is quite essential for the sustainable operation of cloud based systems and also beneficial to intended users. Hence,

***H5:*** *IT infrastructure is positively related to health care professionals’ behavioral intention to use the health cloud*.

### Social influence

Social influence is considered as a construct which a user is being influenced by others to use a specific technology. Previous studies [15] revealed that social influence does not have a significant effect on healthcare service providers’ intention to use the health cloud particularly when UTAUT is used. Therefore,

***H6:*** *Social influence is positively related to health care professionals’ behavioral intention to use the health cloud*.

### Information sharing

Cloud based health platforms provide remote accessibility to the required data and information of patients. In accordance, information sharing, which can occur either internally or externally is a very important construct to be considered. The free flow of information, within cloud based health platforms, inherits efficient use of resources both to service providers and stakeholders [58]. Use of the shared information enhances the behavior of some users since healthcare professionals could have the chance to proffer the right solutions with regard to the patients’ particular condition [59]. Hence,

***H7:*** *Information sharing is negatively related to health care professionals’ behavioral intention to use the health cloud.*

For the purpose of achieving our aim in this research, questionnaire based approach was employed in order to identify the factors which affects health care professionals’ intention to accept and use the cloud based health center. The questionnaire approach is a well-known method to address a large number of issues simultaneously with high response rate. In addition, since answers to questions are scaled and analyzed, it is possible to predict an event that is likely to occur or relationships that exist between variables to have a sustainable plan for the future. The questionnaire shows the demographic data of respondent as well as the kind of health facility the research is carried out. The results will help in explaining the predictors of health care professionals’ adoption of cloud based health center.

## Methods

### Questionnaire design

The study adopted a quantitative research approach to collect data by using a close-ended questionnaire with a seven-point Likert-scale measurement. While in the questionnaire the demographic information of participants were collected, as could be seen in Table 4, the questionnaire was also designed in accordance to extended UTAUT2 constructs. The study presented a modified framework particular to health care information technology acceptance and specific to the circumstances by the research scope; while considering the statement by Venkatesh et al., [10] that, it is important to extend UTAUT2 model by considering different factors and relations specific to the field. The key variables used in the research model is summarized in Table 2.

**Table 2:** Measures of key constructs used

Constructs	Items	Reference
Performance expectancy	PE1- The use of the Cloud based Health Center will enhance a better work efficiency for the professionals.	[60]
	PE2- The use of Cloud Health Center will save the time of both patients and healthcare professionals.	
	PE3- The use of Cloud Based Health Center allows professionals to monitor a patient from a remote location.	
	PE4- Cloud Based Health Center will increase my productivity.	
Cloud based health knowledge	CBHK1- I am aware of worldwide e-health service implementations for health institutions.	[57]
	CBHK2- I have information of Cloud Based Health Center technologies.	
	CBHK3- I am familiar with the technical infrastructure /mobile applications which has to be used.	
	CBHK4- I have made myself familiar with model processes of Cloud Based Health Center, which are relevant to my practice.	
	CBHK5 - I am aware of the changes that comes with cloud based health technologies and how it will affect my daily work life.	
Social influence	SI1 - Your friends, family and colleagues think that Cloud Based Health Center is a useful thing.	[57, 61, 62]
	SI2 - Your friends, family and colleagues thinks that Cloud Based Health Center would improve quality of service.	
	SI3 - Your friends, family and colleague would also use Cloud Based Health Center.	
	SI4 - Do you often discuss the advantages of E-health technologies with friends/family/colleagues?	
	SI5 - My friends, family and colleagues could assist me in the use of Cloud Based Health Center (when available).	
Self-efficacy	SE1 - I feel confident in finding information on a Cloud Based Health center platform.	[57, 63, 64]
	SE2 - I have the necessary skills for using a Cloud Based Health Center application successfully.	
	SE3 - There are available IT experts to train the staff on Cloud Based Health Center.	
	SE4 - I feel confident to work through all interventions that the platform provides me	
Data security	DS1 - I feel apprehensive about using a Cloud Based Health Center in terms of privacy issues.	[57, 65]
	DS2 - There are available, security measures to protect both internal and external communication from unauthorized users.	
	DS3 - There are security measures to communicate accurate information among health institutions.	
	DS4 - There are security measures to communicate accurate information within health institutions.	
IT infrastructure	II1 - Appropriate IT infrastructure are available for Cloud Based Health Center implementations.	[40]
	II2 - Management of healthcare organization creates a favorable environment to support and encourage the usage of information system.	
	II3 - The budget for the planning and funding of infrastructural investment in health institutions is adequate.	
	II4 - There is a service maintenance in place to maintain health institutions’ existing infrastructure.	
	II5 - The existing infrastructure is adequate to support the use of Cloud Based Health Center	
Information sharing	IS1 - It is acceptable if my personal health information is uploaded on the Cloud Based Health Center.	[57, 61, 66]
	IS2 - I am not bothered if my information is shared with other health institution connected to the platform.	
	IS3 - Overemphasis on patient’s privacy protection hinders the necessary flow of information sharing amongst health institutions.	
	IS4 - Information sharing among the units and departments is effective	
Behavioral intention	BI1- I am excited at learning cloud based health system	[67]
	BI2 – I am willing to use cloud health system	

### Sample and data collection

The targeted participants for this research were healthcare professionals from selected healthcare institutions in Benue State Nigeria. Since it is very necessary to get the behavioral intentions of healthcare professionals in the rural communities, the health institutions were grouped into the following categories: Federal Medical Centers (FMC), Teaching Hospitals (TH), General Hospitals (GH), Private Hospitals (PH) and Primary Healthcare Centers (PHC). The institutions were selected from three different locations: Markurdi, Aliade and Otukpo for the sampling. Data was collected from nine hospitals in general. A total of 420 questionnaires were distributed by hand to healthcare professionals in these health institutions and 350 questionnaires were returned which signifies a response rate of 83.33%. However, after careful assessment 50 questionnaires were found to be incomplete as a result of improper filling of the questionnaire or not filling at all and so were discarded. At the end, only 300 questionnaires were found to be available and useful for the analysis. Table 3 presents the health institutions where research was carried.

**Table 3:** Characteristics of surveyed health institutions

Name of health institution	Number of respondents	Gender		Profession of respondents			Hospital specialty
		Male	Female	Doctors	Nurses	Others	
Hospital A	100	41	59	20	20	60	Teaching hospital
Hospital B	100	41	59	28	18	54	Federal
Hospital C	20	11	9	2	5	13	Private
Hospital D	30	16	14	5	7	18	General
Hospital E	15	10	5	2	3	10	Private
Hospital F	10	5	5	1	3	6	Private
Hospital G	5	3	2	1	2	2	Private
Hospital H	5	2	3	–	3	2	Primary Healthcare
Hospital I	15	6	9	1	4	10	Private
Total	300	135	165	60	65	175	

Alphabets A to I represent health institutions visited

Before giving out the questionnaire to the participants, the authors introduced the objectives of the survey and some concepts, such as Cloud Based Health Platform (CBHP), Cloud Based Health Knowledge (CBHK) and Cloud Based Health Center (CBHC), were explained to them. The rationale behind this, was to make sure that participants clearly understand the concepts used in the survey and also, to ensure participants have a prior knowledge of CBHP as the otherwise of this is an exemption.

As could be seen from the Table 3, healthcare professionals who responded to the questionnaire included doctors, nurses, administrative staffs, pharmacists and laboratory technologists. It was also realized that most of the responded participants are female which is contrary of any developing country according to [68]*.* An ethical approval with the reference no MOH/STA/204/VOL 1/28 was given by the Research Ethics Committee of Nigeria.

### Data analysis

SPSS (V20.0) was employed to carry out the statistical analysis, such as construct reliability, convergent validity, discriminant validity and the intraclass correlation coefficient by Fleiss while LISREL (9.30) software was used for the Structural Equation Modeling (SEM) to test the model. Our reason for using this method stem from the fact that SEM is employed to designate the enormous number of models used to assess the validity of basic theories with experimental data. The choice of the LISREL software is as a result of its flexibility in carrying out both structural and measurement components simultaneously. It also presents information referred to as modification indices which aids in the identification of equality constraints. In LISREL, the scales of the observed data is automatically regulated by limiting the loading to one in which there is no need to draw unique factors representing measurement error for each of the observed data. It is believed that the appropriate use of this software will not only provide a statistical basis using a chi-square test for multiple group comparison but can accurately produce good estimates [69]. Composite Reliability (CR), Convergent Validity (Average Variance Extracted - AVE) and correlation coefficient (Fornell-Larcker-Criterion and Intra-class coefficients) analyses were used for the measurement of reliability indices. In addition, path coefficient (β) and associated t- value was used for the analysis of the structural model. Finally, Chi Square value was utilized for the general assessment of the model fit. Prior to testing the structural model, the measurement model was evaluated in order to measure the convergent validity, discriminant validity and correlation coefficient analysis.

## Results

### Demographic analysis

The demographic data of the respondent which shows information such as gender, occupation and years of experience of the respondents are shown in Table 4. The result portrays majority of the respondent to be female (55.0%), while the male respondents sum-up to (45.0%). This is contrary to previous studies [70] in developing countries which has shown marginalization of women in employment. This finding, however, did not affect the research.

**Table 4:** Demographic data of respondent (*n* = 300)

Variable	Category	Frequency (n)	Percentage (%)
Gender	Male	135	45.0
	Female	165	55.0
Occupation of respondents	Doctors	60	20.0
	Nurses	65	21.7
	Others	175	58.3
Years of experience	1–6	205	68.3
	7–12	40	13.3
	13–18	19	6.3
	19–31 above	36	12.0

**Fig. 4:**
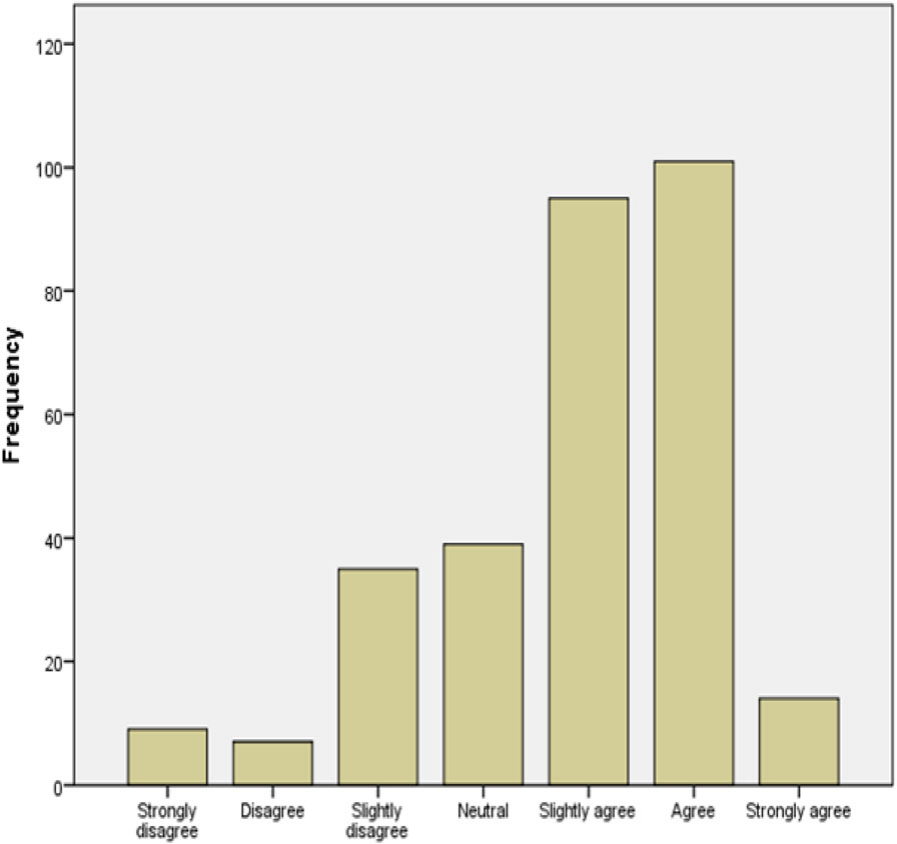
Level of cloud based health knowledge of respondents

One of the other objective of the research is to identify the level of cloud based health knowledge of the healthcare professionals. In accordance, the descriptive analysis for the level of CBHK is depicted in Fig. 4.

It was revealed from Fig. 4 that large number of the respondents (70.1%) agree that they have a good knowledge of cloud based health center. In the same way, just 17% disagree with having a good knowledge of cloud based health center and 13% of the respondent were indecisive. This finding indicates that healthcare professionals have a vibrant knowledge of basic cloud based systems and this finding is a very important step in the acceptance, implementation and usage of the cloud based health center.

### Reliability analysis

Validity and reliability tests of the proposed model’s constructs are first checked by carrying out a general sample test in order to get a meaningful performance measurements before testing the hypothesis. By engaging the average variance extracted (AVE) and composite reliability (CR), the convergent validity was tested [71]*.* Table 5 lists the constructs, item, factor loading, CR and AVE. In order to examine, construct reliability, either the Cronbach Alpha or the composite reliability coefficient (CR) is used. Our testing, as seen on the Table 5, shows composite reliability (CR) value of each of the constructs to be above 0.7 and average variance extracted (AVE) to be above 0.5 respectively. This is the required acceptable threshold value for CR and AVE [72] which indicates a good internal consistency. However, social influence item was removed as the AVE indices (0.4296) are less than the recommended threshold. Table 5 presents the factor loading which is above 0.5 for items associated factors. Indicating adequate reliability according to [73]. The construct reliability of all items is evaluated based on each item reliability. Each item reliability is analyzed by observing the construct loadings [74].

**Table 5:** Composite reliability of dependent and independent variables

Construct	Item	Factor loading	CR	AVE
Performance Expectancy	PE1	0.824	0.841	0.6198
	PE2	0.877		
	PE3	0.650		
	PE4	0.780		
Cloud Based Health Knowledge	CBHK1	0.769	0.893	0.628
	CBHK2	0.818		
	CBHK3	0.848		
	CBHK4	0.836		
	CBHK5	0.681		
Self -Efficacy	SE1	0.860	0.810	0.5267
	SE2	0.814		
	SE3	0.481		
	SE4	0.688		
Data Security	DS1	0.673	0.826	0.545
	DS2	0.804		
	DS3	0.784		
	DS4	0.683		
IT Infrastructure	IT1	0.861	0.9265	0.7163
	IT2	0.777		
	IT3	0.873		
	IT4	0.893		
	IT5	0.823		
Social Influence	SI1	0.750	0.786	(0.4296)
	SI2	0.707		
	SI3	0.670		
	SI4	0.459		
	SI5	0.653		
Information Sharing	IS1	0.739	0.797	0.5035
	IS2	0.841		
	IS3	0.716		
	IS4	0.498		
Behavioral Intention towards CBHS	BI1	0.823	0.821	0.697
	BI2	0.847		

*CR* Composite reliability and the *AVE* Average variance extracted values in bracket are not used in the analysis

### Correlation coefficient analysis

In order to ascertain the level of relationship existing between the independent and dependent variables of the model, correlation coefficient analysis was carried out. Table 6 depicts the square root of AVE and the discriminant validity with respect to the correlation between the constructs at an average range of (0.005 < r < 0.572). Indicating that independent variable can linearly predict dependent variables from the extended UTAUT model with a substantial amount of precision. According to [23], 0.3 and 0.9 are the required threshold value to specify substantial correlation between variables. Indicators illustrates that items load more with individual constructs than with any other construct. Additionally, square root of AVE for each construct is seen to be to be greater than correlations among constructs, indicating discriminant validity for all constructs. This satisfies the Fornell-Larcker-Criterion and is a pointer to the discriminant and convergent validity of the items.

**Table 6:** Discriminant characteristics of constructs

Construct	PE	CBHK	SE	DS	IT	SI	IS	BI
PE	**0.787**							
CBHK	0.224	**0.792**						
SE	0.280	0.484	**0.726**					
DS	0.240	0.494	0.453	**0.738**				
IT	0.019	0.343	0.397	0.355	**0.846**			
SI	0.248	0.453	0.572	0.452	0.462	**0.655**		
IS	0.082	0.259	0.260	0.215	0.170	0.436	**0.710**	
BI	0.005	0.058	0.014	0.037	0.027	0.036	0.044	**0.834**

Note: Square root of AVE in the diagonal (Bold)

In addition, another approach was also considered using intraclass correlation coefficients (ICC) to measure the level of agreement between constructs. In this analysis, ICC estimates and associated 95% confident interval were calculated using a single-rating, absolute-agreement, 2-way-mixed- effects model. Table 7 shows the measurement indices for this analysis. ICC for each single measure was seen to be .195 (*p* < .001). Whereas, average measures for all constructs was seen to be .659 (p < .001), which, according to the recommended values (0.41–0.60) is considered a moderate agreement [74]. Also, the confidence interval for the ICC measure was found to be 95%, ranging between .589 and .719, indicating that, there is a 95% chance the true ICC value is at any point between .589 and .719. Therefore, based on the studies of [75], it could be concluded that substantial agreement do exist among the constructs with moderate reliability.

**Table 7:** Results of ICC evaluation showing Single-Rating and Absolute-Agreement using 2-Way-Mixed effects Model

	Intraclass Correlation	95% Confidence Interval			F Test with Value 0		
		Lower Bound	Upper Bound	Value	*df1*	*df2*	Sig
Single Measures	.195	.152	.243	3.261	299	2093	.001
Average Measures	.659	.589	.719	3.261	299	2093	.001

### Structural model - hypothesis testing and validation of model

**Fig. 5:**
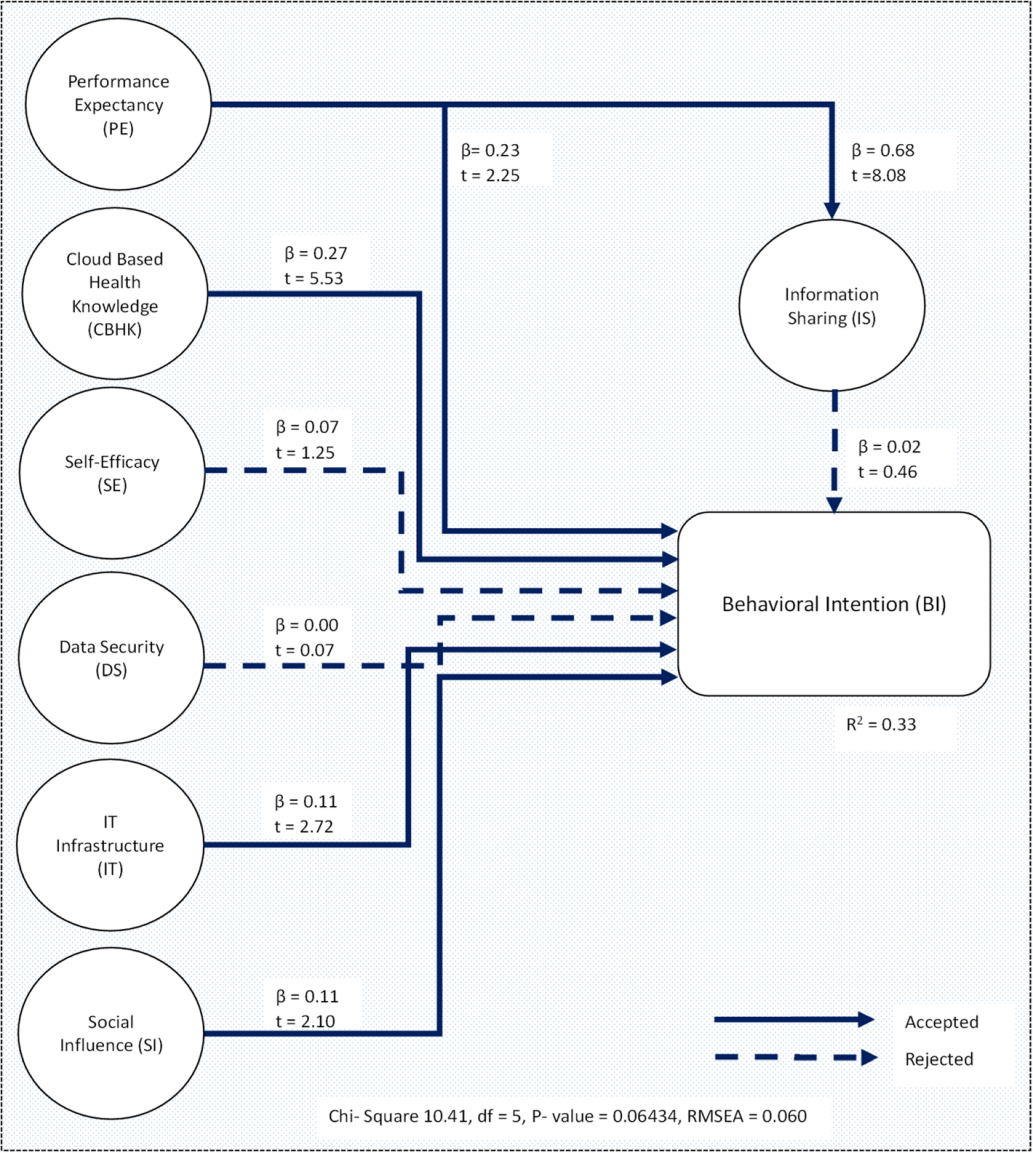
Structural Model

Proposed hypothesis were tested via analysis of the path coefficient between variables and their respective t – values which identifies the significance of the final model. From this analysis, it could be stated that the path coefficients are an indicator for the associated strength of the effect of independent constructs (e.g. performance expectancy, self-efficacy, social influence etc.) on the dependent construct (e.g. behavioral intention). Figure 5 shows the findings of the structural model.

The effect of performance expectancy on information sharing (H1a) and performance expectancy on behavioral intention (H1b) was positive at (β = 0.68; t = 8.08) and (β = 0.23; t = 2.25) as well as the effect of cloud based health knowledge (H2) on behavioral intention (β = 0.27; t = 5.53) respectively going by the recommended criterion given by [66]. Similarly, IT infrastructure (H5) and social influence (H6) both indicate significant effects on behavioral intention (β = 0.11; t = 2.72) (β = 0.11; t = 2.10) respectively. However, self-efficacy (H3), data security (H4) and information sharing (H7) indicated a negative effect and hence, not supported (β = 0.07; t = 1.52), (β = 0.00; t = 0.07) and (β = 0.02; t = 0.46) as they all seem to have a negative effect on behavioral intention. Hence, they were rejected. Summarized results of hypothesis testing is shown in Table 8.

**Table 8:** Summarized results for hypothesis examination

Hypothesis	Path coefficient (β)	T – value	Result
PE IS	0.68	8.08	Accepted
PE BI	0.23	2.25	Accepted
CBHK BI	0.27	5.53	Accepted
SE BI	0.07	1.52	Rejected
DS BI	0.00	0.07	Rejected
IT BI	0.11	2.72	Accepted
SI BI	0.11	2.10	Accepted
IS BI	0.02	0.46	Rejected

The overall model fit was analyzed with LISREL using the values of chi square 10.41. Model fit is derived from dividing chi square by the degree of freedom (DF) (i.e. 10.41/5 = 2.0). Therefore, the model fit was found to be 2.0 and is an acceptable value according to [76] in which chi square should be < = 2 or < = 3. For the fit measure, the Root Mean Square Error of Approximation (RMSEA) was found to be 0.06 which is also within the range of acceptable RMSEA (RMSEA < = .05, RMSEA > = .08), suggesting that the model is supported by statistical testing.

The adequacy seen in the structural model gave room for the evaluation of the explanatory and estimated predictive power. Explanatory power was evaluated by observing the R^2^ value of the dependent variable which defines the number of variance variable explained. R^2^ value for the dependent variable BI (R^2^ = 0.33) was seen to be moderate, elucidating 33.0% variance of the variable. Indicating that, the proposed model reveals 33.0% of behavioral intention.

## Discussion

The objective of this study is to ascertain the issues hindering the adoption of Cloud Based Health Center by healthcare professionals in Nigeria, and at the same time, propose/develop the cloud based health center. Eight variables were used in the study. Four were adopted from the study of Vankatesh et al. 2012 [10] and another four variables (cloud based health knowledge, data security, IT infrastructure and information sharing) integrated from our study area. In line with previous studies on UTAUT2 [10, 31], findings from this study (Fig. 5) reveals that five of the variables have a significant impact on the behavioral intention of healthcare professionals. Performance expectancy (PE) which was our first findings was seen to have a significant effect on information sharing and behavioral intention [10]. This means that information sharing is pivotal and well accepted among healthcare professionals and as such, influences the behavior of healthcare professionals towards the acceptance and use of cloud based health center. Our second finding is the impact of “cloud based health knowledge (CBHK)” on “behavioral intention” while this variable was derived from our study area (Nigeria), it was seen to have a positive impact on the intention of healthcare professionals in Nigeria as they already have a good understanding of cloud based systems (CBS); as such, are willing to accept and use the cloud based health center. Hence, CBHK could be considered as a major driver for creating awareness in CBS for health. Our third finding “self-efficacy” was seen to have a negative impact on behavioral intention. This portrays that a lot of healthcare professionals in Nigeria cannot adequately use CBS on their own and if they are to accept and use cloud based health center, then serious focus should be on training on how to efficiently use CBS independently.

Data security which is our fourth findings was also seen to negatively impact health care professional’s behavioral intention. This seems to be a major barrier to the acceptance and use of cloud based health center among healthcare professionals in Nigeria as security of data is paramount to the survival of any CBS [77]*.* Our fifth and sixth findings “IT infrastructure” and “social influence” all shows positive impacts on the behavioral intention of healthcare professionals. Our last finding “information sharing” however, shows a negative impact on BI. This means that if health care professionals in Nigeria are to accept and use a cloud based health center, then focus should be on providing a mechanism for adequate information sharing within and outside the various departments of health care institutions.

The result of our findings in general portrays CBHK and IT infrastructure to have the highest impact on BI: as such, considered to have significant influence in the acceptance and use of CBHC among healthcare professionals in Nigeria. Our findings, therefore, confirms other UTAUT2 studies in cloud based health systems [78]*.* Our theoretical research model, explain 33% predictive power of healthcare professionals intention to accept the cloud based health center. It could be stated that the model has a significant effect on health care professional’s intention.

Moreover, the study was undertaken to respond to three basic questions:

1. *What is the level of cloud-based health knowledge (CBHK) of healthcare professionals in Nigeria?*

Findings from our research portrays that healthcare professionals have a fair understanding of how a cloud based systems functions confirming the studies of [79, 80] who affirms that health professional’s knowledge of cloud computing platforms indeed have an impact on their attitude and perception towards its adoption and usage.

2. *What are the factors that can militate against the acceptance and use of cloud-based health center (CBHC) in Nigeria?*

Based on related studies of [81] on the adoption of cloud based health systems and also findings from our research, principal factors which militate the acceptance of cloud based health center in Nigeria were recognized and categorized into four which include (1) performance expectancy related to the work efficiency of healthcare professionals, remote access monitoring and increased productivity; (2) cloud based health knowledge related to awareness, technological know-how of healthcare professionals and their knowledge, experience; (3) IT infrastructure related to technical support, software/hardware and favorable work environment; (4) social influence related to quality of service and the necessity of the platform.

3. *How can Nigeria adopt and use the CBHC?*

Our research findings indicate that healthcare professionals are ready and willing to adopt CBHC however, there is a low availability of required IT infrastructure for the sustainability of CBHC. Therefore, more emphasis should be placed on the provision of needed ICT tools and technology. This finding corresponds to the study of [82] who identified information communication as a bedrock to the implementation of any cloud based systems. In addition, more awareness/sensitivity needs to be created as regard the relevance of CBHC in the Nigerian health care system as indicated from the research findings.

Factors and dynamics are identified which signify positive impact on healthcare service providers’ behavioral intention to accept the CBHC in Nigeria. Having identified these factors enhance successful and sustainable CBHC implementations in the near future which could benefit healthcare professionals from excessive work related stress and offers distant access to health care delivery services.

The limitations of this study are as follows: It is possible for selection bias to take place since all the respondents are health workers; their knowledge and awareness of cloud based health systems could be more versatile than that of a non-health worker. The choice of constructs used here is based on our observation of our selected study site; it is therefore possible that other constructs of cloud health usage may exist. In addition, this study cannot be generalized since it targets only health care workers. Future work could be conducted to determine health care consumers adoption of cloud based health center utilizing constructs that really focus on patients’ needs. Finally, some limitations could arise from the use of CBHC, one of which is the inability of rural dwellers to independently use the system. However, this limitation could be minimized since there is the HCW to educate and guide them on how the system functions. Secondly, the password could easily be stolen since most of the users are likely to use their mobile phones. In addition, internet access might not be readily available to the public, except users subscribe to individual internet service from service providers who are well represented in the communities.

## Conclusion

The main focus of this research study is to identify dynamics for the adoption and the implementation of cloud based systems in the Nigerian health care system and the necessity of CBHC. Fundamental factors were primarily identified which affect the adoption of cloud based health center from the perspective of healthcare professionals in Nigerian hospitals. Our research findings is of managerial importance to policy makers in that our model explain 33% of the factors which hinders the successful implementation of a cloud based health system in Nigeria and contributes to the sustainability and growth of the Nigerian health care system.

## Abbreviations

AVE: Average Variance Extracted
BI: Behavioral intention
CBHC: Cloud Based Health Center
CBHK: Cloud based health knowledge
CBHP: Cloud based health platform
CBHS: Cloud based health system
CBT: Cloud based technologies
CDs: Compact Disc
CR: Composite Reliability
DS: Data security
FMC: Federal Medical Center
GH: General Hospital
GS: General satisfaction
HCW: Healthcare worker
ICC: Intraclass Coefficient
ICT: Information Communication Technology
IS: Information sharing
IT: Information technology
ITU: International Telecommunication Union
NCC: Nigerian Communication Commission
PE: Performance expectancy
PEOU: Perceived ease of use
PH: Private Hospital
PHC: Primary Healthcare Center
PU: Perceive Usefulness
SE: Self-efficacy
SEM: Structural Equation Model
SHC: Secondary health care
SI: Social influence
TAM: Technology Acceptance Model
TH: Teaching Hospital
THC: Tertiary health care
UTAUT: Unified Theory of Acceptance and Use of Technology
UTAUT2: Unified Theory of Acceptance and Use of Technology Two
β: Beta
